# Unbiased Gene Expression Analysis Implicates the huntingtin Polyglutamine Tract in Extra-mitochondrial Energy Metabolism

**DOI:** 10.1371/journal.pgen.0030135

**Published:** 2007-08-17

**Authors:** Jong-Min Lee, Elena V Ivanova, Ihn Sik Seong, Tanya Cashorali, Isaac Kohane, James F Gusella, Marcy E MacDonald

**Affiliations:** 1 Center for Human Genetic Research, Massachusetts General Hospital, Boston, Massachusetts, United States of America; 2 Children's Hospital Informatics Program, Children's Hospital, Boston, Massachusetts, United States of America; 3 Huntington's Disease Society of America Coalition for the Cure Mitochondria and Energy Metabolism Team, New York, New York, United States of America; University of Minnesota, United States of America

## Abstract

The Huntington's disease (HD) CAG repeat, encoding a polymorphic glutamine tract in huntingtin, is inversely correlated with cellular energy level, with alleles over ∼37 repeats leading to the loss of striatal neurons. This early HD neuronal specificity can be modeled by respiratory chain inhibitor 3-nitropropionic acid (3-NP) and, like 3-NP, mutant huntingtin has been proposed to directly influence the mitochondrion, via interaction or decreased PGC-1α expression. We have tested this hypothesis by comparing the gene expression changes due to mutant huntingtin accurately expressed in *STHdh^Q111/Q111^* cells with the changes produced by 3-NP treatment of wild-type striatal cells. In general, the HD mutation did not mimic 3-NP, although both produced a state of energy collapse that was mildly alleviated by the PGC-1α-coregulated nuclear respiratory factor 1 (Nrf-1). Moreover, unlike 3-NP, the *HD* CAG repeat did not significantly alter mitochondrial pathways in *STHdh^Q111/Q111^* cells, despite decreased *Ppargc1a* expression. Instead, the HD mutation enriched for processes linked to huntingtin normal function and Nf-κB signaling. Thus, rather than a direct impact on the mitochondrion, the polyglutamine tract may modulate some aspect of huntingtin's activity in extra-mitochondrial energy metabolism. Elucidation of this *HD* CAG-dependent pathway would spur efforts to achieve energy-based therapeutics in HD.

## Author Summary

Huntington's disease (HD) is a tragic neurodegenerative disorder caused by a CAG repeat that specifies the size of a glutamine tract in the huntingtin protein, such that the longer the tract, the earlier the loss of striatal brain cells. A correlation of polyglutamine tract size has also implicated huntingtin in the proper functioning of mitochondria, the cell's energy factories. Here we have tested the prevailing hypothesis, that huntingtin may directly affect the mitochondrion, by using comprehensive gene expression analysis to judge whether the HD mutation may replicate the effects of 3-nitropropionic acid (3-NP), a compound known to inhibit mitochondria, with loss of striatal neurons. We found that, while mutant huntingtin and 3-NP both elicited energy starvation, the gene responses to the HD mutation, unlike the responses to 3-NP, did not highlight damage to mitochondria, but instead revealed effects on huntingtin-dependent processes. Thus, rather than direct inhibition, the polyglutamine tract size appears to modulate some normal activity of huntingtin that indirectly influences the management of the mitochondrion. Understanding the precise nature of this extra-mitochondrial process would critically guide efforts to achieve effective energy-based therapeutics in HD.

## Introduction

The CAG trinucleotide repeat in the Huntington's disease gene *(HD)* is highly polymorphic in humans, with alleles ranging from ∼6 to >100 units encoding a variable polyglutamine tract in huntingtin, a large (>350 kDa) HEAT domain protein [1]. The gene was discovered because alleles over ∼35–37 units, whether inherited in one copy or, in rare cases, two copies, are associated with the onset of Huntington's disease (HD) symptoms, including dance-like movements, cognitive decline, and psychiatric disturbance [1]. This intriguing disease-initiating mechanism, while relatively insensitive to dosage, is exquisitely progressive with allele size, such that the age at onset of HD symptoms is progressively decreased as CAG length is increased [1].

The early HD pathology comprises the selective loss of medium-size spiny neurons in the striatum that forms the HD pathological grading system [2]. The ability of mitochondrial respiratory chain poisons such as succinate dehydrogenase inhibitor 3-nitropropionic acid (3-NP) to produce HD-like striatal-specific cell loss has implied a role for mitochondrial dysfunction in HD pathogenesis [3]. Indeed, numerous studies have demonstrated that deficits in measures of energy metabolism become manifest in presymptomatic and symptomatic HD brain and peripheral tissues [4–11].

Investigations of the early consequences of the *HD* CAG repeat in human lymphoblastoid cell lines have recently implicated the polyglutamine tract size in huntingtin in modulating cellular ATP/ADP ratio across the entire non-HD and HD range [12]. The longest alleles were associated with the lowest ATP/ADP ratios, while alleles in the non-HD range were associated with progressively higher energy levels [12]. Moreover, consistent with a role for the polyglutamine tract in influencing an intrinsic huntingtin function in energy metabolism, the targeted deletion of the short seven-glutamine tract from murine huntingtin yielded elevated cellular ATP, with early senescence, and improved motor performance in *Hdh* ^ΔQ/ΔQ^ mice [13].

The shared downstream consequences of the *HD* CAG tract and 3-NP have implied that the polyglutamine tract in huntingtin, like 3-NP, may directly affect the mitochondrion [14]. Huntingtin is detected throughout the cell, in the nucleus and in the cytoplasm, where it can associate with mitochondria [15], implicating a direct “toxic” interaction [15–17]. However, recent findings, including studies in *STHdh^Q111/Q111^* striatal cells, with a knock-in juvenile onset CAG repeat accurately expressed as endogenous huntingtin with 111 glutamines [18], suggested that mutant huntingtin may influence mitochondrial biogenesis/function by decreasing *Ppargc1a* transcription [19,20]. This gene encodes peroxisome proliferative activated receptor gamma, coactivator 1 alpha (PGC-1α), a key cofactor for Nrf-1 and other mitochondrial transcription regulators.

To probe the earliest consequences of the *HD* CAG mechanism, we tested the mitochondrial hypothesis by using unbiased gene expression analysis to monitor the extent to which the accurate expression of the *HD* CAG repeat, in *STHdh^Q111/Q111^* striatal cells, may reproduce the consequences of 3-NP challenge. The results confirmed a shared downstream energy collapse but did not indict direct 3-NP-like effects of the *HD* CAG repeat on the mitochondrion. Instead, the data have elevated the candidacy of extra-mitochondrial pathways in huntingtin modulation of energy metabolism.

## Result/Discussion

We and others have demonstrated previously that *STHdh^Q111/Q111^* cells, compared to wild-type *STHdh^Q7/Q7^* cells (expressing endogenous seven-glutamine huntingtin), exhibited mitochondrial energy phenotypes similar to the effects of 3-NP treatment, including decreased mitochondrial respiration [21] and ATP synthesis [12,21,22]. However, *STHdh^Q111/Q111^* cells also have been shown to display phenotypes opposite to the effects reported for 3-NP challenge, such as increased, instead of decreased, levels of the reactive oxygen species (ROS) scavenger glutathione [23], suggesting that 3-NP might not precisely mimic the effects of the HD mutation.

**Figure 1:**
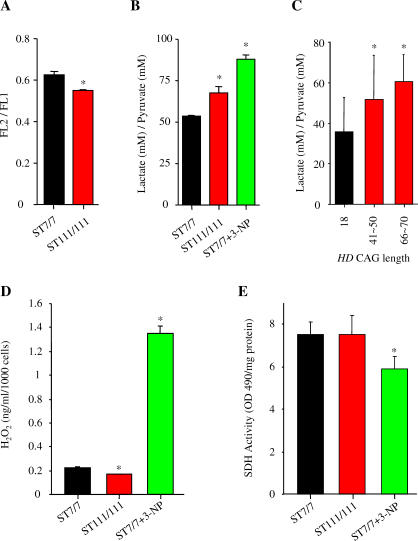
Mitochondrial Phenotypes Altered by the HD Mutation or 3-NP Treatment (A) Mitochondrial membrane potential, denoted by the FL1/FL2 ratio, was significantly decreased by the HD mutation in ST*Hdh^Q111/Q111^* cells (ST111/111), compared to wild-type ST*Hdh^Q7/Q7^* cells (ST7/7). (B) Lactate/pyruvate ratio was significantly increased in both ST*Hdh^Q111/Q111^* cells (ST111/111) and 3-NP treated wild-type ST*Hdh^Q7/Q7^* cells (ST7/7 + 3-NP), compared to untreated ST*Hdh^Q7/Q7^* cells (ST7/7). (C) Lactate/pyruvate ratio was significantly increased in HD lymphoblastoid cells as the length of the longer of the two *HD* CAG alleles was increased, from the typical normal size (18 CAG) to the adult onset HD (42–50 CAG) and juvenile onset HD (66–70) ranges. (D) ROS, monitored by hydrogen peroxide concentration, was significantly decreased in ST*Hdh^Q111/Q111^* cells (ST111/111) compared to ST*Hdh^Q7/Q7^* cells (ST7/7) but was dramatically increased in wild-type cells following treatment with 3-NP. (E) Succinate dehydrogenase activity was similar in ST*Hdh^Q111/Q111^* cells (ST111/111) and ST*Hdh^Q7/Q7^* cells (ST7/7), but was significantly decreased by 3-NP treatment of wild-type ST*Hdh* ^Q7/Q7^ cells (ST7/7 + 3-NP). In all panels, asterisk denotes significant difference (*p* < 0.05) from ST7/7 by Student *t*-test.

To explore this notion, we examined additional energy phenotypes and observed, as reported for 3-NP challenge, that the HD mutation decreased mitochondrial membrane potential (Figure 1A) and elevated the lactate/pyruvate ratio (Figure 1B) indicative of altered energy homeostasis. Indeed, in human lymphoblastoid cells, this ratio was increased in severity with *HD* CAG repeat size (Figure 1C), demonstrating that mitochondrial dysfunction is likely to be a consequence of the *HD* CAG size-dependent mechanism. However, while 3-NP, as reported [24], was associated with increased ROS, as measured by hydrogen peroxide levels, the *STHdh^Q111/Q111^* cells, compared to wild-type cells, exhibited decreased ROS (Figure 1D). Moreover, while 3-NP treated wild-type cells, as expected, displayed decreased succinate dehydrogenase activity, the longer *HD* CAG repeat expressed in the *STHdh^Q111/Q111^* cells did not significantly change the activity of this respiratory chain component (Figure 1E). Thus, while superficially similar, the energy phenotypes displayed by mutant striatal cells did not in detail recapitulate the effects of 3-NP challenge, implying distinct underlying pathways.

To delineate the effects of the *HD* CAG repeat and 3-NP treatment, without making a priori assumptions about the underlying biology, we performed global analysis to monitor the expression of the mitochondrial and the nuclear genomes. As sequences for genes located on the mitochondrial genome were not represented on the murine Affymetrix MG 430 2.0 arrays microarrays, which we used to analyze the nuclear genome (below), the expression of nine of the 13 mitochondrial genes encoding respiratory components was assessed using specific RT-PCR assays. The mRNA levels for each of these genes was dramatically reduced in 3-NP treated cells, compared to untreated wild-type striatal cells, whereas mRNA levels did not differ significantly between *STHdh^Q111/Q111^* and wild-type cells (Figure S1). Thus, consistent with unchanged succinate dehydrogenase activity, mutant huntingtin did not reproduce the effects of 3-NP on the mitochondrial genome, implying that the energy deficits in *STHdh^Q111/Q111^* cells might instead stem from altered expression of the nuclear genes that regulate the mitochondrion.

**Figure 2:**
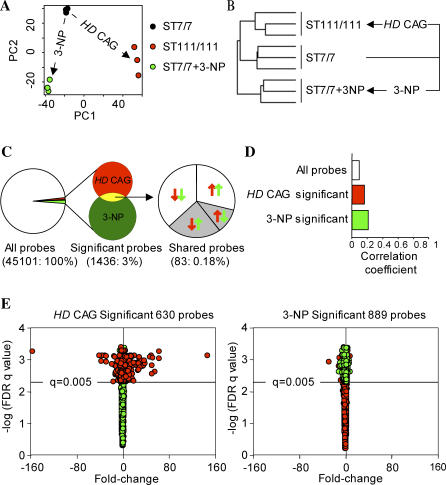
*HD* CAG Mutation and 3-NP Treatment Produced Distinct Signatures (A) Plot of unsupervised principal component analysis of triplicate datasets for wild-type striatal cells (ST7/7/), mutant striatal cells (ST111/111), and wild-type cells treated with 3-NP (ST7/7+3-NP), revealing discrete gene expression space for the *HD* CAG repeat (comparison of ST111/111 and ST7/7) and 3-NP treatment (comparison of ST7/7+3-NP and ST7/7). (B) Dendrogram plot, generated by average linkage clustering algorithm analysis of the expression values of all probes, indicated proper clustering of triplicate samples within each group and revealed that the mutant cell group (ST111/111) was more closely related to the wild-type cell group (ST7/7) than to the 3-NP treated wild-type cell samples (ST7/7+3-NP). Arrows depict the data set comparisons to generate changes due to the HD CAG and 3-NP treatment. (C) Diagram summarizing the identification of significantly altered probe signals using a stringent FDR *q*-value 0.005, which revealed 630 probes different between mutant and wild-type striatal cells (HD CAG) and 889 probes changed between wild-type cells and wild-type cells treated with 3-NP (3-NP), with 83 probes significant in both comparisons (shared). Of these, 70 % were concordantly changed. (D) Plot of the correlation coefficients between the *HD* CAG- and 3-NP-changes for all probes (white), for the *HD* CAG significant probes (red), and for the 3-NP significant probes (green), demonstrating the distinct nature of the changes in the two comparisons. (E) Volcano plots of fold-change versus FDR *q-*value for probes significant (*q* < 0.005) in the *HD* CAG comparison (red) and those same probes in the 3-NP comparison (green) (left panel) or the converse (right panel), showing the small overlap and more dynamic expression changes for the *HD* CAG repeat, compared to 3-NP treatment.

We then performed unbiased analysis of the nuclear genome expression datasets to determine the extent to which the consequences of the *HD* CAG repeat may mirror the effects of 3-NP challenge. The results of principle component (Figure 2A) and cluster analysis (Figure 2B) of all probes demonstrated that, whereas the replicate datasets were highly related, the wild-type, 3-NP treated, and mutant cell datasets were quite distinct. Thus, rather than giving rise to similar effects, which may differ in magnitude, the consequences of the *HD* CAG repeat and 3-NP appeared to be fundamentally different. Indeed, at a stringent false discovery rate (FDR) (*q* < 0.005), approximately the same proportion of all probes (3%) was significantly altered either by the *HD* CAG repeat mutation (comparing mutant versus wild-type cells) (Table S1) or by 3-NP challenge (comparing 3-NP treated wild-type versus wild-type cells) (Table S2), with the *HD* CAG yielding some changes of larger magnitude (fold-change) than 3-NP. However, little overlap was detected between the two probe lists (summarized in Figure 2C and 2E), yielding low correlation coefficients between *HD* CAG and 3-NP changed probes (Figure 2D). Indeed, for the most part, these represented different genes, which are plotted by chromosome in Figure S2. Thus, while the impact, in terms of number and magnitude of changes, was similar, HD mutation did not reproduce the molecular effects of 3-NP.

**Figure 3:**
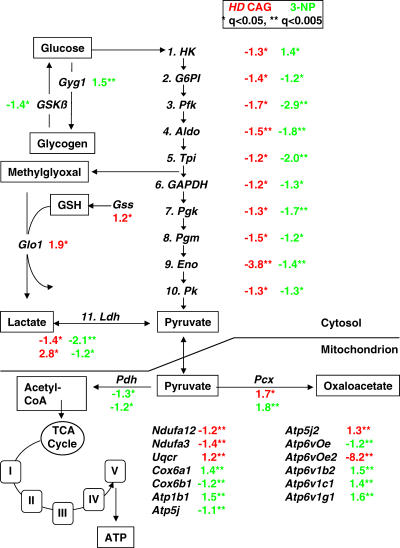
*HD* CAG and 3-NP Shared Decreased Expression of Glycolytic Genes The energy metabolism pathway gene expression changes common to mutant striatal cells (*HD* CAG, red) and 3-NP treated wild-type striatal cells (3-NP, green) are shown, with fold-change, compared to wild-type striatal cells, highlighting decreased cytosolic glycolytic processes (numbered 1–11). The HD mutation but not 3-NP treatment was associated with increased expression of *Ldh2* and of enzymes in the formation and breakdown of glutathione or the toxic product methylglyoxyal. Single asterisk denotes FDR *q*-value <0.05 double asterisks denote FDR *q-*value < 0.005.

**Figure 4:**
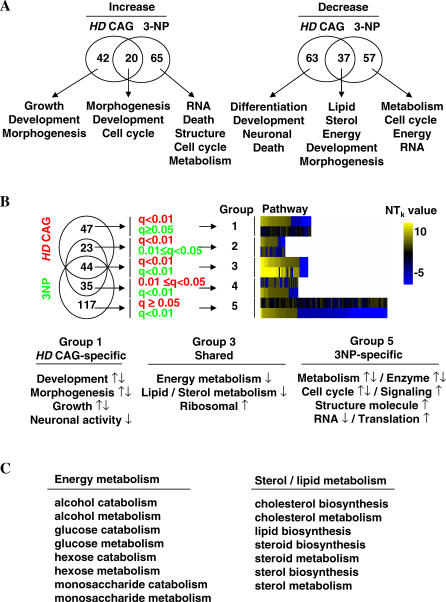
Pathways Common to *HD* CAG and 3-NP Reveal Decreased Carbohydrate and Lipid Metabolism, with *HD* CAG-Specific Pathways Reminiscent of huntingtin Normal Function (A) Venn diagrams summarize the results of GO analysis (biological process), showing the numbers of significantly altered (Increase, Decrease) *HD* CAG-specific, 3-NP-specific and shared GO terms, with the major categories of pathways listed below. (B) Venn diagram, statistical criteria, and NT_k_ value-based heatmap summarize the results of sigPathway analysis to distinguish gene sets that were *HD* CAG specific (Group 1), 3-NP specific (Group 5), or highly significant in both the *HD* CAG and 3-NP comparisons (Group 3). Groups 2 and 4 were judged marginally significant. High absolute NT_k_ value indicates high significance and +NT_k_ and –NT_k_ denote enrichment in the control group (ST7/7) and the experimental group (ST111/111 or ST7/7+3-NP), respectively. The major categories of enriched gene sets, with the direction of change denoted by arrows, are listed. (C) A list of the 15 pathways that were found to be common to the HD mutation and 3-NP treatment, as judged significant in both GO and sigPathway analysis. All were enriched in wild-type cells (ST7/7) compared to mutant striatal cells or 3-NP treated wild-type cells and denoted glycolysis and lipid/sterol metabolism.

The mitochondrial hypothesis predicted that both the HD mutation and 3-NP treatment would alter mitochondrial energy genes, prompting an examination of the small fraction (0.18%) of all probes significantly altered by both insults. These represented 83 known genes (plotted by chromosome in Figure S3 and listed in Table S3), which did not highlight the mitochondrion but instead spotlighted decreased cytosolic energy production (Figure 3). More sensitive tests of groups of genes in functional pathways, using the Gene Ontology (GO) biological process (Figure 4A) and sigPathway gene set analysis [25] (Figure 4B), confirmed that the HD mutation and 3-NP were both associated with highly significant decreases in carbohydrate metabolism and, as reported previously [26], lipid (sterol/cholesterol) biosynthesis (Figure 4C). Thus, striatal cells, unlike glia or other cell types, may possess a limited capacity to adjust glycolytic flux in response to changes in mitochondrial ATP synthesis [27], thereby providing a potential explanation for the ability of 3-NP to mimic the early loss of striatal neurons in HD striatum.

Although the notion that the HD mutation and 3-NP might commonly alter the expression of nuclear encoded mitochondrial genes did not appear to be borne out, inspection of the data did reveal significant changes in a few mitochondria-related energy genes in mutant striatal cells, which, notably, were unchanged by 3-NP (Figure 3). *Ppargc1a* (encoding PGC-1α) was decreased, as reported previously [19]. Mitochondrial components in the transfer electrons from NADH to the respiratory chain *(Ndufa12* and *Ndufa3)* or in the transport of hydrogen ions *(Atp6v0e2)* were decreased. By contrast, mRNAs encoding the iron sulfur-binding factor of complex III *(Uqcr)* and a nonenzymatic component of the ATP synthase complex *(Atp5j2)* were elevated. Moreover, consistent with elevated glutathione [23], the expression of genes that detoxify free radical derivatives *(Ldh2, Gss,* and *Glo1)* was increased, suggesting that altered redox state may contribute distinctly to low energy (and lipid) metabolism in mutant striatal cells.

**Figure 5:**
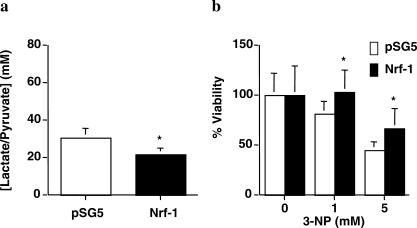
Nrf-1 Ameliorated Mutant Striatal Cell Lactate/Pyruvate and Survival (A) Bar plot of lactate/pyruvate ratio in ST*Hdh^Q111/Q111^* striatal cells transiently transfected with pSG5 control plasmid (white) or Nrf-1 expression plasmid (black), demonstrating improvement with the latter. Asterisk denotes *p* < 0.05 by Student's *t*-test. (B) Bar plot of viability of ST*Hdh^Q111/Q111^* cells that were transiently transfected with control pSG5 plasmid (white) or Nrf-1 plasmid (black), challenged with various concentrations of 3-NP, showing increased survival with the latter. Asterisk denotes *p* < 0.05 by Student's *t*-test.

The decrease in *Ppargc1a* mRNA implied that mitochondrial regulatory transcription factors such as Nrf-1, which are coregulated by PGC-1α, might be expected to improve energy metabolism in mutant cells. Indeed, over-expression of Nrf-1 in *STHdh^Q111/Q111^* cells altered the mRNA levels of known Nrf-1 target genes (Table S4) but, as reported for PGC-1α [19], only mildly improved both cellular lactate/pyruvate ratio (Figure 5A) and cell survival following respiratory chain inhibition (Figure 5B). Thus, coupled with the apparent dearth of changes in mitochondrial factors, the modest effects of boosting Nrf-1 strongly suggested that mutant huntingtin may not directly perturb the mitochondrion.

**Figure 6:**
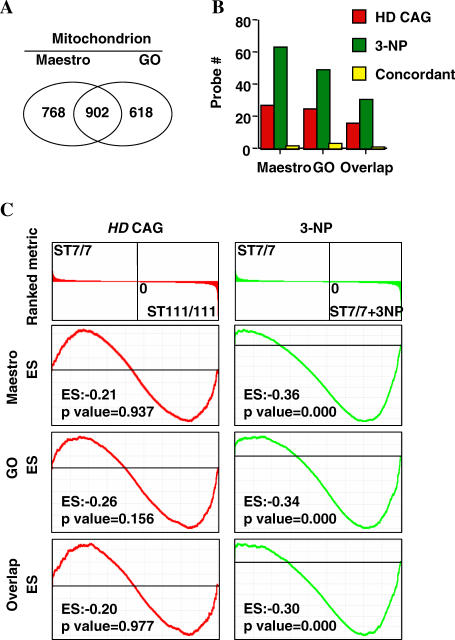
3-NP but Not the HD mutation altered Nuclear-Encoded Mitochondrial Gene Expression (A) Venn diagram depicting the numbers of probes in two overlapping nuclear gene sets, either annotated by GO cellular component (GO) or predicted by MAESTRO [28] to encode mitochondrial gene products, with those identified in both procedures forming a third gene set (Overlap). (B) Bar plot of probe number percentage versus FDR *q*-value for each of the mitochondrial probe sets (MAESTRO, GO, overlap) revealed that fewer mitochondrial components were altered in the *HD* CAG comparison (red) than in the 3-NP comparison (green), with only a small subset concordantly altered by both insults (yellow). (C) Plots of GSEA ranked metric and ES revealed that in each case the mitochondrial gene sets were significantly enriched only in wild-type cells treated with 3-NP (ST7/7+3-NP) and not in mutant striatal cells (ST111/111), compared to wild-type striatal cells (ST7/7).

Consequently, to more rigorously examine this possibility, we used gene set enrichment analysis (GSEA) to specifically determine whether mitochondrial pathways or processes may be perturbed by 3-NP or the HD mutation. GSEA examines, as a group, genes that form a functional pathway, thereby capturing small effect sizes that when considered in single gene analyses may not have reached our stringent statistical threshold. We tested two sets of ∼1,500 nuclear genes that were either GO annotated or predicted by the integrative genomics program MAESTRO [28] to encode mitochondrial products, as well as a third set comprising the group of 902 genes that were common to both lists (Table S5; Figure 6A and 6B). The GSEA results, plotted as enrichment scores in Figure 6C, demonstrated that, compared to wild-type cells, each of the gene sets was highly enriched by 3-NP treatment. In striking contrast, no significant enrichment of any of the mitochondrial gene sets was detected in *STHdh^Q111/Q111^* cells. Thus, these findings, which were consistent with the results of the mitochondrial genome analysis (Figure S1), clearly revealed that the HD mutation did not reproduce the direct effects of 3-NP on the mitochondrion. Therefore, contrary to predictions from decreased *Ppargc1a* mRNA or interactions of mutant huntingtin with the mitochondrion [14–17,19,20], our results consistently implied that the *HD* CAG mechanism may primarily influence energy metabolism via extra-mitochondrial cellular pathways.

Since our data supported the view that the processes by which the HD mutation and 3-NP may lead to energy starvation were largely distinct, we reasoned that the pathways that did mediate the effects of the huntingtin polyglutamine tract would be those that were not affected by 3-NP challenge. To support this approach, we tested whether the early presymptomatic consequences of the *HD* CAG repeat in medium-size spiny striatal neurons in vivo might be recapitulated by accurate expression of the expanded *HD* CAG repeat in cultured *STHdh^Q111/Q111^* cells or by 3-NP challenge of wild-type cells. Microarray analysis of mRNA from medium-size spiny striatal neurons, obtained by laser capture microscopy (LCM) from post-mortem brain, has been reported [29]. The major class of LCM genes judged to be the most significantly altered by the *HD* CAG in that study, all with decreased expression, yielded a set of 38 mouse genes (Table S14) that were tested by GSEA in our striatal cell *HD* CAG and 3-NP datasets. The results demonstrated that this human LCM gene set was significantly decreased in the *STHdh^Q111/Q111^* cells (enrichment score [ES] 0.51, *p-*value 0.000, FDR *q-*value 0.180) but was not altered in the 3-NP treated cells (ES 0.38, *p-*value 0.271, FDR *q-*value 0.713). Thus, the early molecular consequences of the *HD* CAG repeat in striatal neurons in human brain also become manifest as a consequence of accurate expression of the expanded repeat in the cultured *STHdh^Q111/Q111^* cells but these were not reproduced by 3-NP challenge. This supported the approach of attempting to identify the processes by which huntingtin may modulate energy metabolism by examination of *HD* CAG repeat–specific changes.

Therefore, stringent statistical filtering criteria were used to identify those gene sets that were enriched in mutant striatal cells but not in 3-NP treated wild-type cells (Tables S6–S12). Remarkably, a major class of pathways uniquely enriched in *STHdh^Q111/Q111^* cells, summarized in Figure 4A and 4B, pointed to processes implicated in huntingtin's essential normal activities [30–33]: development/morphogenesis (central nervous system, mesoderm, and embryo); growth, cell motility, migration, and locomotion; cell adhesion; neuronal processes; and TGF-β and BMP signaling. A minor class, which denoted increased gap junction channel (connexin), phosphate, and anion transport, may also prove to be related to huntingtin function. These results, therefore, strongly implicated huntingtin normal function in the capacity of the polyglutamine tract to influence mitochondrial function and cellular energy metabolism in *STHdh^Q111/Q111^* cells.

Notably, this finding is consistent with a genetic gain-of-function hypothesis for the mechanism that initiates the HD pathogenic process. The polyglutamine tract size may progressively influence energy homeostasis by increasing some intrinsic huntingtin activity. Alternatively, it may capitalize on an opportunity afforded by huntingtin function to modulate an unrelated cellular component.

What might this normal huntingtin activity be? One possibility, inferred from previous work with huntingtin-deficient cells [34,35], as well as with *STHdh^Q111/Q111^* cells [18], may reflect a role for huntingtin in intracellular iron trafficking. However, huntingtin's normal activities impact a broad range of cellular processes, from vesicle trafficking to the proper regulation of gene transcription [33,36,37]. As a starting point, therefore, we reasoned that worthy candidates for huntingtin modulation of energy metabolism might be found among the transcriptional regulators that most frequently orchestrate the gene changes in *STHdh^Q111/Q111^* cells, but not 3-NP treated cells.

**Figure 7:**
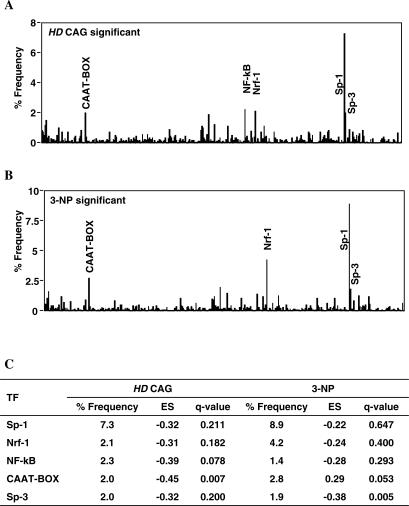
Transcription Factor Analysis to Identify *HD* CAG-Specific Regulators (A) A plot of the normalized frequency of putative transcription factor binding sites identified from MAPPER database, for genes altered by the HD mutation (*HD* CAG). (B) A plot of the normalized frequency of putative transcription factor binding sites identified from MAPPER database, for genes altered by 3-NP treatment (3-NP). (C) Results of GSEA of candidate transcription factors that may frequently regulate target genes altered in mutant, compared to wild-type, striatal cells (*HD* CAG) or 3-NP treated, compared to untreated wild-type, striatal cells (3-NP), showing ES and FDR *q-*value statistic.

Unbiased transcription factor analysis coupled with GSEA yielded five *HD* CAG–specific candidates (Figure 7). Interestingly, NRSF/REST, associated with altered expression of genes in *STHdh^Q111/Q111^* cells and in HD brain [37], did not emerge as a candidate, and NRSF/REST target genes or genes with upstream RE1 binding sites were not enriched as a consequence of the *HD* CAG repeat (unpublished data). The top candidates for *HD* CAG specific regulators were CAAT-Box (e.g., NF-Y, cEBP, and CTF) and nuclear factor kappa B (Nf-κB), with weaker support for Nrf-1 and Sp-1. Most of these candidates, including Nrf-1, have been shown to exert extra-mitochondrial effects on energy metabolism, affecting, e.g., fatty oxidation, glycolysis, glutathione synthesis, and cell stress-sensing [38–40]. Moreover, by associating exclusively with mutant huntingtin (or fragment), PGC-1α, a coregulator with Nrf-1, Sp-1, and its coregulator TAF4, as well as core components of Pol II transcriptional machinery (TFIID, TFIIF) have been implicated in HD pathogenesis [19,41–43].

However, the mechanism by which the *HD* CAG modulates cellular energy levels was manifest even at non-HD-causing lengths, in the cadre of normal huntingtin function [12]. Nf-κB/Dorsal/RelB has been linked previously to huntingtin's normal function [44]. Nf-κB was a modifier of huntingtin carboxyl terminal fragment–induced phenotypes in *Drosophila* [44]. Furthermore, endogenous normal human huntingtin can associate with the p50 subunit of Nf-κB [44], which binds to target gene promoters in a redox-dependent manner [45]. This raises a new hypothesis that merits investigation in a variety of suitable polymorphic *HD* CAG systems, namely, that huntingtin polyglutamine tract length may influence Nf-κB-mediated signaling, perhaps via redox sensing, either as a cause or a consequence of modulating energy metabolism.

In summary, the results of this study demonstrated that the early (presymptomatic) consequences of the juvenile onset *HD* CAG allele, accurately expressed in *STHdh^Q111/Q111^* striatal cells, did not in most details mimic mitochondrial respiratory chain inhibitor 3-NP, although both metabolic challenges produced a common response—the collapse of mitochondrial and cytosolic energy processes. This reveals the limited utility of 3-NP lesion models for uncovering the pathways by which the polyglutamine tract in huntingtin may influence energy metabolism or for prioritizing agents that may modify these processes. Indeed, the data uniformly refute the widely held view of a direct mutant huntingtin-specific effect on the mitochondrion. Instead, our data implicate an effect of the polyglutamine tract on some normal activity of the huntingtin protein in extra-mitochondrial energy metabolism, perhaps redox sensing.

It is interesting to note that redox sensing cell signaling, via ROS-dependent pathways, is emerging as a regulator of glucose and lipid metabolism in health, aging, and disease [46]. If huntingtin proves to be involved, the molecules and gene products that modify oxidation-sensitive signaling in metabolic disorders may become candidates for modifying huntingtin-regulated mitochondrial metabolism in HD. By the same token, agents that modify redox sensing signaling in HD may also be of interest in cancer and a variety of complex metabolic disorders.

## Materials and Methods

### Cell culture and 3-NP treatment.

ST*Hdh^Q7/Q7^* and ST*Hdh^Q111/Q111^* cells, generated from striatal primordia of wild-type *Hdh^Q7/Q7^* and homozygous mutant *Hdh^Q111/Q111^* knock-in mouse embryos, respectively [18], were cultured in DMEM (33 °C, 5% CO_2_, 10% FBS, and 400 μg/ml G418). Challenge of ST*Hdh^Q7/Q7^* cells with 3-NP (1 mM) was for 48 h. Previously genotyped human lymphoblastoid cell lines (*HD* CAG 17/18, 15/42, 21/43, 21/66, 19/70, 41/48, and 45/50) were cultured in RPMI-1640 (37 °C, 5% CO_2_, and 5% FBS).

### Mitochondrial membrane potential, succinate dehydrogenase activity, ROS, and lactate/pyruvate ratio.

Mitochondrial membrane potential was measured by incubation of cells with JC-1 (2.5 μg/ml) for 20 min at 33 °C, followed by flow cytometry (FACScalibur, http://www.bdbiosciences.com/), counting 10^4^ cells in the FL1 (monomer) and FL2 (aggregate) channels and calculating FL2/FL1 ratio.

Succinate dehydrogenase activity, measured using succinate as a substrate and 3-(4,5-dimethylthiazol-2-yl)-5-(3-carboxymethoxyphenyl)-2-(4-sulfophenyl)-2H-tetrazolium as an electron acceptor, was normalized by protein concentration [47]. ROS levels were monitored by determinations of hydrogen peroxide concentration, using a chemiluminescent hydrogen peroxide detection kit (Assay Designs, http://www.assaydesigns.com/). Data were quantified compared to a standard curve, and normalized to cell number. Lactate and pyruvate concentrations in cleared lysates (3% perchloric acid, sonication) were determined by HPLC analysis (Aminex column, Bio-Rad Laboratories, http://www.bio-rad.com/; 0.6 ml/min 0.05 mM H_2_SO_4_; UV detection at 210 nm) using appropriate standards. All measurements derive from triplicate experiments, using duplicate samples. Data are given as the mean, +/− one standard deviation.

### Mitochondrial genome gene expression analysis.

Reverse transcription PCR (RT-PCR) analysis for the genes listed in Table S5 was performed (Bio-Rad iCycler) with gene-specific primer sets listed in Table S13. The ΔΔC_T_ method was used to calculate gene expression levels, compared to β-actin control [48].

### Microarray analysis of nuclear genome gene expression.

Total RNA (5 μg), isolated from triplicate cell cultures, was converted using SuperScript II reverse transcriptase (Invitrogen. http://www.invitrogen.com) to labeled cRNA, and 25 μg of labeled probe was hybridized to Affymetrix MG 430 2.0 arrays (http://www.affymetrix.com). Expression data was generated and normalized using RMA [49] and significant probes were identified by FDR (*q* < 0.005) [50]. A selection of genes judged to be significantly altered according to these criteria was tested by semi-quantitative RT-PCR analysis (primer sets in Table S13), with ∼80% (13 of 16 randomly chosen genes) concordant with the microarray results. For pathway analysis, significant (*p* < 0.05) GO terms in significantly altered probe sets were identified by GO analysis (DAVID 2006) [51] and, using all probes, significantly enriched (*q* < 0.01) pathways were identified by permutation-based GSEA (sigPathway) [25]. For the GSEA comparison of the mouse and human presymptomatic striatal cell changes, the LCM gene set comprised the 38 most robust LCM genes, all decreased by the *HD* CAG repeat, drawn from the table in Hodges et al. [29], that could be unambiguously mapped to a corresponding mouse locus. Cluster [52], dChip [53], and Bioconductor [54] were used for clustering and data visualization.

### Nrf-1 transfection and cytotoxicity assay.

ST*Hdh^Q111/Q111^* cells were transiently transfected with control vector (pSG5) or Nrf-1 mammalian cell expression plasmid (0.2 μg of plasmid/well) using lipofectamine as described by the manufacturer (Invitrogen). At 48 h post-transfection, lactate/pyruvate ratios were determined, as described above, and viability following 3-NP challenge was measured by MTS cytotoxicity assay (Promega, http://www.promega.com/). Nrf-1 activity was judged by measuring the expression of a selection of Nrf-1 target genes (Table S4), using semi-quantitative RT-PCR analysis with gene-specific primer sets (Table S13).

### Transcription factor analysis.

For significant genes, putative transcription factor binding sites were identified from the MAPPER database [55], by searching 500 bp upstream from the transcription initiation site and then selecting the top ten transcription factors, by score, for each gene. A total of 4,400 hits (358 transcription factors) and 6,684 hits (371 transcription factors) were recorded for *HD* CAG and 3-NP treatment, respectively, and the frequency for each was then calculated relative to the total number of hits. As a statistical test, the 100 top target genes for each transcription factor that exhibited 2% or higher frequency in each comparison were identified and used in the transcription factor analysis, to build a custom set for gene set enrichment analysis [56].

## Supporting Information

Figure S1 Mitochondrially Encoded Gene Expression Levels Bar plot of mRNA levels, determined in RT-PCR assays, for nine mitochondrially encoded genes encoding respiratory chain polypeptides in wild-type striatal cells (ST7/7), mutant striatal cells (ST111/111), and wild-type striatal cells treated with 3-NP (ST7/7+3-NP), revealing decreased expression with 3-NP but not the HD mutation.
(33 KB PPT)

Figure S2 *HD* CAG and 3-NP Mostly Alter the Expression of Different Genes The significant (FDR *q* < 0.005) gene changes associated with the HD mutation in mutant versus wild-type striatal cells (*HD* CAG) or with 3-NP treatment of wild-type cells versus untreated wild-type cells (3-NP) are depicted by chromosomal location and can be viewed in detail using probe ID (Tables S1 and S2) and dChip software [53].
(1.4 MB PPT)

Figure S3 Genes Altered by Both the *HD* CAG and 3-NP Treatment The shared significant (FDR *q* < 0.005) gene changes associated with the HD mutation in mutant versus wild-type striatal cells (*HD* CAG) or with 3-NP treatment of wild-type cells versus untreated wild-type cells (3-NP) are depicted by chromosomal location and can be viewed in detail using probe ID (Table S3) and dChip software.
(477 KB PPT)

Table S1 Probes Significantly Altered by *HD* CAG (122 KB XLS)

Table S2 Probes Significantly Altered by 3-NP Treatment (182 KB XLS)

Table S3 Probes Significantly Altered by *HD* CAG and 3-NP Treatment (30 KB XLS)

Table S4 Nrf-1 Target Gene Expression (30 KB DOC)

Table S5 Nuclear Genes Encoding Mitochondrial Components (275 KB XLS)

Table S6 Shared Significant GO Terms by *HD* CAG and 3-NP Comparison (47 KB DOC)

Table S7 *HD* CAG-Specific GO Terms (45 KB DOC)

Table S8 3-NP-Specific GO Terms (147 KB DOC)

Table S9 Shared Gene Sets by *HD* CAG and 3-NP (Group 3) (33 KB DOC)

Table S10 *HD* CAG-Specific Gene Sets (Group 1) (34 KB DOC)

Table S11 3-NP-Specific Gene Sets (Group 5) (165 KB DOC)

Table S12 Significant Pathways Identified by Both GO Analysis and Gene Set Analysis (60 KB DOC)

Table S13 Primer Sets for Gene-Specific PCR Assays (28 KB DOC)

Table S14 LCM-Derived Gene Set (22 KB XLS)

### Accession Numbers

MIAME compliant microarray data were deposited at the Gene Expression Omnibus (GEO, http://www.ncbi.nlm.nih.gov/geo/) with Accession number GSE3583.

## Abbreviations

ES: enrichment score
FDR: false discovery rate
GSEA: gene set enrichment analysis
GO: gene ontology
HD: Huntington's disease
*HD,*: Huntington's disease gene
LCM: laser capture microscopy
3-NP: 3-nitropropionic acid
ROS: reactive oxygen species
RT-PCR: reverse transcription PCR
