# Supplementary material for: Unbiased Gene Expression Analysis Implicates the huntingtin Polyglutamine Tract in Extra-mitochondrial Energy Metabolism
Source: PLoS Genet. 2007 Aug 17;3(8):e135. doi: 10.1371/journal.pgen.0030135 (PMC1950164; doi:10.1371/journal.pgen.0030135)
Supplement: Supplementary file 1 [file pgen.0030135.st004.doc]

**Supplementary Table 4. Nrf-1-target gene expression**

Gene expression changes of some of Mapper-identified Nrf-1 target genes in *HD* CAG and 3-NP model were listed. To check functional expression of Nrf-1 target genes, total RNA was isolated following transfection with Nrf-1 plasmid, and expression levels of these genes were measured by reverse transcription and real-time PCR.

| Gene | Accession | *HD* CAG | | 3-NP | | RT-PCR |
| --- | --- | --- | --- | --- | --- | --- |
|  |  | Fold | q-value | Fold | q-value | Fold |
| Abhd5 | AK007421 | -1.2 | 0.1567 | -1.2 | 0.0589 | 1.5 |
| Adssl1 | NM_007421 | 1.5 | 0.0269 | 1.3 | 0.0500 | 1.7 |
| Akap8 * | BG069776 | 1.1 | 0.2548 | 1.2 | 0.0137 | 1.0 |
| Arpc1a * | NM_019767 | 1.4 | 0.0441 | 1.0 | 0.3678 | 1.1 |
| Atp6v1g1 * | BI154058 | -1.1 | 0.0453 | 1.3 | 0.0041 | 1.3 |
| Cxcr4 | D87747 | -1.0 | 0.4519 | -1.1 | 0.1455 | 1.2 |
| Dctn6 | NM_011722 | 1.1 | 0.1804 | 1.0 | 0.3058 | 1.4 |
| Hspca | BM243794 | -1.3 | 0.0257 | 1.1 | 0.1268 | -1.0 |
| Nfib | BB242844 | -1.0 | 0.5998 | -1.1 | 0.2535 | 1.5 |
| Rpl10a * | AK002613 | 1.0 | 0.4094 | 1.1 | 0.1035 | -1.0 |
| Sfrs2 * | AF250135 | -1.2 | 0.1225 | -1.3 | 0.0518 | 1.2 |
| Smad5 | U77638 | 1.4 | 0.0715 | 1.1 | 0.2243 | 1.2 |
| Stag1 | BG071268 | 1.0 | 0.5054 | -1.0 | 0.2011 | -1.0 |
| Uble1a | BB293365 | 1.0 | 0.5458 | 1.1 | 0.2621 | 1.4 |

* Identified as Nrf-1 target genes by location analysis in quiescent human T98G cells.
