# Supplementary material for: Unbiased Gene Expression Analysis Implicates the huntingtin Polyglutamine Tract in Extra-mitochondrial Energy Metabolism
Source: PLoS Genet. 2007 Aug 17;3(8):e135. doi: 10.1371/journal.pgen.0030135 (PMC1950164; doi:10.1371/journal.pgen.0030135)
Supplement: Supplementary file 2 [file pgen.0030135.st006.doc]

**Supplementary Table 6. Shared significant GO terms by *HD* CAG and 3-NP comparison**

Significant probes in *HD* CAG (630 probes, q<0.005) and those in 3-NP comparison (889 probes, q<0.005) were used for GO analysis (DAVID 2006) to identify commonly significant GO terms (biological process, p<0.05) in *HD* CAG and 3-NP model. Among 284 significant GO terms, 57 were shared by *HD* CAG and 3-NP model (*HD* CAG, p<0.05 and 3-NP, p<0.05). %, % of probes with defined gene ontology term in the significant probes. p value, Fisher’s exact test p value.

| **Shared GO Term by *HD* CAG and 3-NP: Increase** | *HD* CAG | | 3-NP | |
| --- | --- | --- | --- | --- |
|  | % | p value | % | p value |
| **Cell Growth, Cycle** |  |  |  |  |
| cell proliferation | 4.5 | 0.0016 | 2.6 | 0.0439 |
| regulation of cell cycle | 3.5 | 0.0205 | 3.1 | 0.0014 |
| regulation of cell growth | 2.6 | 0.0001 | 1.0 | 0.0334 |
| regulation of progression through cell cycle | 3.5 | 0.0201 | 3.1 | 0.0013 |
| **Development, Morphogenesis** |  |  |  |  |
| blood vessel morphogenesis | 1.9 | 0.0370 | 1.3 | 0.0446 |
| cellular morphogenesis | 4.2 | 0.0062 | 3.3 | 0.0021 |
| development | 15.0 | 0.0002 | 11.0 | 0.0050 |
| morphogenesis | 10.9 | 0.0000 | 5.7 | 0.0095 |
| **Others** |  |  |  |  |
| angiogenesis | 1.9 | 0.0184 | 1.3 | 0.0171 |
| cellular physiological process | 49.5 | 0.0006 | 50.6 | 0.0000 |
| cellular process | 60.4 | 0.0003 | 54.7 | 0.0111 |
| metabolism | 36.4 | 0.0223 | 37.6 | 0.0000 |
| negative regulation of progression through cell cycle | 1.6 | 0.0462 | 1.1 | 0.0262 |
| positive regulation of biological process | 5.1 | 0.0415 | 4.7 | 0.0048 |
| positive regulation of cellular physiological process | 4.8 | 0.0114 | 4.0 | 0.0029 |
| positive regulation of cellular process | 4.8 | 0.0224 | 4.4 | 0.0012 |
| positive regulation of physiological process | 5.1 | 0.0075 | 4.1 | 0.0029 |
| regulation of cellular physiological process | 19.5 | 0.0006 | 14.7 | 0.0295 |
| regulation of cellular process | 20.1 | 0.0007 | 15.4 | 0.0259 |
| regulation of physiological process | 20.5 | 0.0003 | 15.1 | 0.0327 |

| **Shared GO Term by *HD* CAG and 3-NP: Decrease** | *HD* CAG | | 3-NP | |
| --- | --- | --- | --- | --- |
|  | % | p value | % | p value |
| **Energy** |  |  |  |  |
| alcohol catabolism | 1.5 | 0.045 | 2.6 | 0.000 |
| alcohol metabolism | 4.9 | 0.000 | 7.3 | 0.000 |
| gluconeogenesis | 1.1 | 0.036 | 1.3 | 0.005 |
| glucose catabolism | 1.5 | 0.042 | 2.6 | 0.000 |
| glucose metabolism | 2.3 | 0.007 | 2.9 | 0.000 |
| hexose catabolism | 1.5 | 0.043 | 2.6 | 0.000 |
| hexose metabolism | 2.3 | 0.019 | 2.9 | 0.001 |
| monosaccharide catabolism | 1.5 | 0.043 | 2.6 | 0.000 |
| monosaccharide metabolism | 2.3 | 0.020 | 2.9 | 0.001 |
| pyruvate metabolism | 1.1 | 0.047 | 1.3 | 0.008 |
| **Lipid, Sterol** |  |  |  |  |
| carboxylic acid metabolism | 4.5 | 0.018 | 5.1 | 0.002 |
| cellular lipid metabolism | 5.3 | 0.003 | 8.6 | 0.000 |
| cholesterol biosynthesis | 2.3 | 0.000 | 4.1 | 0.000 |
| cholesterol metabolism | 2.3 | 0.001 | 4.1 | 0.000 |
| fatty acid metabolism | 2.3 | 0.033 | 2.6 | 0.006 |
| lipid biosynthesis | 4.2 | 0.000 | 7.3 | 0.000 |
| lipid metabolism | 6.4 | 0.001 | 9.2 | 0.000 |
| steroid biosynthesis | 3.0 | 0.000 | 5.4 | 0.000 |
| steroid metabolism | 3.0 | 0.001 | 5.4 | 0.000 |
| sterol biosynthesis | 2.6 | 0.000 | 4.5 | 0.000 |
| sterol metabolism | 2.6 | 0.000 | 4.5 | 0.000 |
| **Development, Morphogenesis** |  |  |  |  |
| blood vessel morphogenesis | 3.8 | 0.000 | 1.9 | 0.048 |
| development | 23.0 | 0.000 | 12.4 | 0.046 |
| morphogenesis | 13.2 | 0.000 | 7.0 | 0.024 |
| organ development | 13.6 | 0.000 | 7.0 | 0.030 |
| organ morphogenesis | 9.4 | 0.000 | 4.5 | 0.025 |
| **Others** |  |  |  |  |
| angiogenesis | 3.0 | 0.000 | 1.9 | 0.024 |
| biosynthesis | 8.7 | 0.020 | 13.4 | 0.000 |
| cellular physiological process | 52.1 | 0.001 | 60.5 | 0.000 |
| cellular process | 62.3 | 0.010 | 63.7 | 0.002 |
| negative regulation of biological process | 7.6 | 0.001 | 6.7 | 0.005 |
| negative regulation of cellular physiological process | 6.0 | 0.004 | 5.7 | 0.004 |
| negative regulation of cellular process | 7.6 | 0.001 | 5.7 | 0.020 |
| negative regulation of physiological process | 6.0 | 0.006 | 6.1 | 0.003 |
| organic acid metabolism | 4.5 | 0.018 | 5.1 | 0.002 |
| regulation of angiogenesis | 1.1 | 0.039 | 1.3 | 0.006 |
| cell organization and biogenesis | 13.2 | 0.004 | 12.7 | 0.005 |
