# Supplementary material for: Unbiased Gene Expression Analysis Implicates the huntingtin Polyglutamine Tract in Extra-mitochondrial Energy Metabolism
Source: PLoS Genet. 2007 Aug 17;3(8):e135. doi: 10.1371/journal.pgen.0030135 (PMC1950164; doi:10.1371/journal.pgen.0030135)
Supplement: Supplementary file 3 [file pgen.0030135.st007.doc]

**Supplementary Table 7. *HD* CAG-specific GO terms**

Significantly increased (349 probes, q<0.005) and decreased probes (281 probes, q<0.005) in *HD* CAG comparison were used for GO analysis (DAVID 2006) to identify significant GO terms (biological process, p<0.05) in *HD* CAG model. Among 162 significant GO terms, 105 GO terms were *HD* CAG-specific (*HD* CAG, p<0.05 and 3-NP, p≥0.05). %, % of probes with defined gene ontology term in the significant probes. p value, Fisher’s exact test p value.

| ***HD* CAG-specific GO Term: Increase** | % | p value |
| --- | --- | --- |
| anion transport | 3.2 | 0.001 |
| behavior | 2.9 | 0.021 |
| cell adhesion | 6.4 | 0.000 |
| cell cycle arrest | 1.3 | 0.018 |
| cell growth | 3.2 | 0.000 |
| chemotaxis | 1.9 | 0.012 |
| embryonic development | 3.5 | 0.006 |
| embryonic development (sensu Metazoa) | 1.9 | 0.031 |
| embryonic morphogenesis | 2.2 | 0.008 |
| enzyme linked receptor protein signaling pathway | 3.8 | 0.000 |
| growth | 3.5 | 0.000 |
| inflammatory response | 2.9 | 0.001 |
| inorganic anion transport | 3.2 | 0.000 |
| locomotory behavior | 2.6 | 0.006 |
| negative regulation of biological process | 6.7 | 0.002 |
| negative regulation of cellular metabolism | 2.6 | 0.040 |
| negative regulation of cellular physiological process | 5.4 | 0.005 |
| negative regulation of cellular process | 6.4 | 0.002 |
| negative regulation of metabolism | 2.9 | 0.034 |
| negative regulation of organismal physiological process | 1.3 | 0.023 |
| negative regulation of physiological process | 5.8 | 0.003 |
| negative regulation of signal transduction | 1.9 | 0.003 |
| negative regulation of transcription from RNA polymerase II promoter | 1.6 | 0.043 |
| negative regulation of transcription, DNA-dependent | 1.9 | 0.048 |
| nucleoside metabolism | 1.3 | 0.008 |
| organ development | 9.0 | 0.000 |
| organ morphogenesis | 6.1 | 0.000 |
| pattern specification | 2.9 | 0.003 |
| phosphate transport | 3.2 | 0.000 |
| regulation of biological process | 21.7 | 0.000 |
| regulation of cell proliferation | 3.2 | 0.006 |
| regulation of cell size | 3.2 | 0.000 |
| regulation of growth | 2.6 | 0.001 |
| regulation of organismal physiological process | 2.6 | 0.021 |
| regulation of signal transduction | 2.9 | 0.004 |
| regulation of transcription from RNA polymerase II promoter | 3.8 | 0.002 |
| response to external stimulus | 5.1 | 0.008 |
| response to wounding | 4.2 | 0.015 |
| skeletal development | 2.6 | 0.002 |
| taxis | 1.9 | 0.012 |
| transcription from RNA polymerase II promoter | 4.2 | 0.003 |
| transmembrane receptor protein tyrosine kinase signaling pathway | 3.2 | 0.000 |

| ***HD* CAG-specific GO Term: Decrease** | % | p value |
| --- | --- | --- |
| Apoptosis | 4.5 | 0.039 |
| axon guidance | 2.3 | 0.002 |
| Axonogenesis | 2.3 | 0.019 |
| blood vessel development | 3.8 | 0.000 |
| brain development | 2.3 | 0.011 |
| cell adhesion | 9.1 | 0.000 |
| cell death | 4.9 | 0.029 |
| cell development | 3.4 | 0.031 |
| cell differentiation | 9.8 | 0.000 |
| cell fate commitment | 1.9 | 0.019 |
| cell migration | 6.0 | 0.000 |
| cell motility | 6.0 | 0.000 |
| cell-cell adhesion | 2.3 | 0.050 |
| cellular morphogenesis | 5.7 | 0.000 |
| cellular morphogenesis during differentiation | 3.0 | 0.005 |
| central nervous system development | 2.6 | 0.006 |
| death | 4.9 | 0.031 |
| embryonic development | 3.4 | 0.024 |
| enzyme linked receptor protein signaling pathway | 3.0 | 0.019 |
| epithelial cell differentiation | 1.1 | 0.044 |
| establishment of localization | 18.9 | 0.012 |
| forebrain development | 1.5 | 0.023 |
| hormone secretion | 1.1 | 0.024 |
| localization | 18.9 | 0.014 |
| localization of cell | 6.0 | 0.000 |
| locomotion | 6.0 | 0.000 |
| morphogenesis of an epithelium | 1.9 | 0.014 |
| motor axon guidance | 1.1 | 0.001 |
| muscle development | 2.3 | 0.009 |
| negative regulation of cellular metabolism | 3.4 | 0.008 |
| negative regulation of metabolism | 3.8 | 0.006 |
| negative regulation of nucleobase, nucleoside, nucleotide and nucleic acid metabolism | 2.6 | 0.039 |
| negative regulation of transcription | 2.6 | 0.031 |
| negative regulation of transcription from RNA polymerase II promoter | 2.3 | 0.007 |
| negative regulation of transcription, DNA-dependent | 2.6 | 0.008 |
| nervous system development | 8.3 | 0.000 |
| neurite morphogenesis | 2.6 | 0.010 |
| neurogenesis | 4.5 | 0.000 |
| neuron development | 2.6 | 0.026 |
| neuron differentiation | 3.4 | 0.008 |
| neuron morphogenesis during differentiation | 2.6 | 0.010 |
| positive regulation of biological process | 6.8 | 0.003 |
| positive regulation of cellular physiological process | 5.3 | 0.011 |
| positive regulation of cellular process | 6.0 | 0.004 |
| positive regulation of physiological process | 6.0 | 0.003 |
| programmed cell death | 4.5 | 0.044 |
| regulation of biological process | 21.1 | 0.008 |
| regulation of cell migration | 1.5 | 0.023 |
| regulation of cell motility | 1.5 | 0.026 |
| regulation of cellular physiological process | 18.9 | 0.011 |
| regulation of cellular process | 20.0 | 0.007 |
| regulation of development | 2.6 | 0.023 |
| regulation of locomotion | 1.5 | 0.030 |
| regulation of metabolism | 14.3 | 0.042 |
| regulation of physiological process | 19.6 | 0.009 |
| regulation of transcription from RNA polymerase II promoter | 3.8 | 0.010 |
| response to external stimulus | 5.3 | 0.016 |
| system development | 9.1 | 0.000 |
| tissue development | 2.6 | 0.040 |
| transcription from RNA polymerase II promoter | 4.5 | 0.003 |
| tube development | 2.6 | 0.002 |
| tube morphogenesis | 2.3 | 0.002 |
| vasculature development | 4.5 | 0.000 |
