# Supplementary material for: Unbiased Gene Expression Analysis Implicates the huntingtin Polyglutamine Tract in Extra-mitochondrial Energy Metabolism
Source: PLoS Genet. 2007 Aug 17;3(8):e135. doi: 10.1371/journal.pgen.0030135 (PMC1950164; doi:10.1371/journal.pgen.0030135)
Supplement: Supplementary file 4 [file pgen.0030135.st008.doc]

# Supplementary Table 8. 3-NP-specific GO terms

Significantly increased (556 probes, q<0.005) and decreased probes (333 probes, q<0.005) in 3-NP comparison were used for GO analysis (DAVID 2006) to identify significant GO terms (biological process, p<0.05) in 3-NP model. Among 179 significant GO terms, 122 GO terms were 3-NP-specific (*HD* CAG, p≥0.05 and 3-NP, p>0.05). %, % of probes with defined gene ontology term in the significant probes. p value, Fisher’s exact test p value.

| **3-NP-specific GO Term: Increase** | % | p value |
| --- | --- | --- |
| actin cytoskeleton organization and biogenesis | 1.3 | 0.048 |
| amine biosynthesis | 1.0 | 0.010 |
| amine metabolism | 3.0 | 0.001 |
| amino acid and derivative metabolism | 2.7 | 0.001 |
| amino acid metabolism | 2.0 | 0.013 |
| Apoptosis | 4.0 | 0.001 |
| biopolymer catabolism | 1.7 | 0.048 |
| biopolymer metabolism | 15.6 | 0.000 |
| biopolymer modification | 9.5 | 0.001 |
| Biosynthesis | 9.8 | 0.000 |
| blood vessel development | 1.4 | 0.040 |
| cell cycle | 4.8 | 0.001 |
| cell death | 4.1 | 0.002 |
| cell division | 1.7 | 0.016 |
| cell organization and biogenesis | 12.0 | 0.000 |
| cellular biosynthesis | 9.1 | 0.000 |
| cellular localization | 4.2 | 0.021 |
| cellular macromolecule metabolism | 17.4 | 0.000 |
| cellular metabolism | 35.1 | 0.000 |
| cellular protein metabolism | 16.8 | 0.000 |
| coenzyme biosynthesis | 1.3 | 0.012 |
| coenzyme metabolism | 1.7 | 0.019 |
| cofactor biosynthesis | 1.4 | 0.007 |
| cofactor metabolism | 1.8 | 0.019 |
| cytoplasm organization and biogenesis | 2.0 | 0.000 |
| Death | 4.1 | 0.002 |
| establishment of cellular localization | 4.1 | 0.031 |
| establishment of localization | 14.6 | 0.044 |
| intracellular signaling cascade | 5.8 | 0.008 |
| Localization | 14.7 | 0.041 |
| macromolecule biosynthesis | 5.7 | 0.000 |
| macromolecule metabolism | 22.6 | 0.000 |
| mitotic cell cycle | 1.7 | 0.021 |
| mRNA processing | 1.6 | 0.038 |
| nitrogen compound biosynthesis | 1.0 | 0.010 |
| nitrogen compound metabolism | 3.0 | 0.002 |
| nucleotide biosynthesis | 1.8 | 0.001 |
| nucleotide metabolism | 2.4 | 0.000 |
| organelle organization and biogenesis | 6.5 | 0.001 |
| phosphate metabolism | 5.9 | 0.001 |
| phosphorus metabolism | 5.9 | 0.001 |
| Phosphorylation | 5.0 | 0.002 |
| physiological process | 54.6 | 0.000 |
| positive regulation of programmed cell death | 1.4 | 0.028 |
| primary metabolism | 33.0 | 0.000 |
| programmed cell death | 4.0 | 0.002 |
| protein amino acid phosphorylation | 4.5 | 0.001 |
| protein biosynthesis | 4.8 | 0.000 |
| protein metabolism | 17.4 | 0.000 |
| protein modification | 9.3 | 0.000 |
| purine nucleotide biosynthesis | 1.3 | 0.002 |
| purine nucleotide metabolism | 1.3 | 0.004 |
| purine ribonucleotide biosynthesis | 1.1 | 0.005 |
| purine ribonucleotide metabolism | 1.1 | 0.009 |
| response to protein stimulus | 0.9 | 0.030 |
| response to unfolded protein | 0.9 | 0.030 |
| ribonucleotide biosynthesis | 1.1 | 0.008 |
| ribonucleotide metabolism | 1.1 | 0.015 |
| ribosome biogenesis | 1.8 | 0.000 |
| ribosome biogenesis and assembly | 1.8 | 0.000 |
| RNA metabolism | 3.1 | 0.005 |
| RNA processing | 2.4 | 0.013 |
| small GTPase mediated signal transduction | 2.1 | 0.021 |
| Translation | 1.6 | 0.021 |
| vasculature development | 1.4 | 0.044 |

| **3-NP-specific GO Term: Decrease** | % | p value |
| --- | --- | --- |
| alcohol biosynthesis | 1.3 | 0.009 |
| biopolymer metabolism | 16.2 | 0.008 |
| carbohydrate biosynthesis | 1.9 | 0.007 |
| carbohydrate catabolism | 2.6 | 0.000 |
| carbohydrate metabolism | 5.1 | 0.001 |
| carboxylic acid biosynthesis | 1.6 | 0.010 |
| cell cycle | 7.0 | 0.000 |
| cell division | 4.1 | 0.000 |
| cellular biosynthesis | 7.3 | 0.038 |
| cellular carbohydrate catabolism | 2.6 | 0.000 |
| cellular carbohydrate metabolism | 3.8 | 0.002 |
| cellular macromolecule catabolism | 2.9 | 0.048 |
| cellular metabolism | 45.2 | 0.000 |
| Chromatin assembly | 2.6 | 0.000 |
| Chromatin assembly or disassembly | 2.6 | 0.001 |
| chromosome organization and biogenesis | 3.8 | 0.006 |
| chromosome organization and biogenesis (sensu Eukaryota) | 3.8 | 0.004 |
| coenzyme metabolism | 2.6 | 0.018 |
| cofactor metabolism | 2.9 | 0.011 |
| cytokinesis | 1.3 | 0.003 |
| DNA metabolism | 4.5 | 0.048 |
| DNA packaging | 4.1 | 0.000 |
| establishment and/or maintenance of chromatin architecture | 3.8 | 0.000 |
| fatty acid biosynthesis | 1.6 | 0.009 |
| generation of precursor metabolites and energy | 6.4 | 0.002 |
| glycolysis | 2.2 | 0.000 |
| hexose biosynthesis | 1.3 | 0.009 |
| isoprenoid biosynthesis | 1.6 | 0.000 |
| isoprenoid metabolism | 1.6 | 0.000 |
| M phase | 2.9 | 0.005 |
| M phase of mitotic cell cycle | 2.2 | 0.010 |
| macromolecule catabolism | 3.5 | 0.016 |
| macromolecule metabolism | 27.4 | 0.000 |
| metabolism | 48.7 | 0.000 |
| microtubule-based process | 2.2 | 0.045 |
| Mitosis | 2.2 | 0.010 |
| mitotic cell cycle | 2.9 | 0.006 |
| monosaccharide biosynthesis | 1.3 | 0.009 |
| mRNA metabolism | 2.9 | 0.011 |
| mRNA processing | 2.6 | 0.016 |
| negative regulation of enzyme activity | 1.3 | 0.014 |
| nuclear mRNA splicing, via spliceosome | 1.9 | 0.031 |
| nucleosome assembly | 2.6 | 0.000 |
| Organelle organization and biogenesis | 8.3 | 0.003 |
| organic acid biosynthesis | 1.6 | 0.010 |
| physiological process | 63.1 | 0.000 |
| primary metabolism | 44.0 | 0.000 |
| protein complex assembly | 3.2 | 0.000 |
| protein metabolism | 18.2 | 0.016 |
| regulation of cell cycle | 5.1 | 0.000 |
| regulation of progression through cell cycle | 5.1 | 0.000 |
| RNA metabolism | 3.8 | 0.027 |
| RNA processing | 3.5 | 0.011 |
| RNA splicing | 2.2 | 0.011 |
| RNA splicing, via transesterification reactions | 1.9 | 0.031 |
| RNA splicing, via transesterification reactions with bulged adenosine as nucleophile | 1.9 | 0.031 |
| Transition metal ion homeostasis | 1.3 | 0.012 |
