# Supplementary material for: Unbiased Gene Expression Analysis Implicates the huntingtin Polyglutamine Tract in Extra-mitochondrial Energy Metabolism
Source: PLoS Genet. 2007 Aug 17;3(8):e135. doi: 10.1371/journal.pgen.0030135 (PMC1950164; doi:10.1371/journal.pgen.0030135)
Supplement: Supplementary file 5 [file pgen.0030135.st009.doc]

**Supplementary Table 9. Shared gene sets by *HD* CAG and 3-NP model (Group 3)**

Significantly enriched gene sets in *HD* CAG and 3-NP comparison were identified by gene set analysis (sigPathway). 114 and 196 gene sets were significantly enriched in *HD* CAG and 3-NP model, respectively (q<0.01). Among 266 significant gene sets, 44 gene sets were shared by *HD* CAG and 3-NP model (*HD* CAG, q<0.01 and 3-NP, q<0.01). Size, number of probes in the gene set. NT_k_, normalized gene set score obtained by gene set permutation.

| **Group 3. *HD* CAG-3-NP Common** **Gene Set** | Size | NT_k_ | |
| --- | --- | --- | --- |
|  |  | *HD* CAG | 3-NP |
| **Energy** |  |  |  |
| Alcohol catabolism | 101 | 6.9 | 11.9 |
| Alcohol metabolism | 392 | 8.5 | 12.5 |
| Carbohydrate catabolism | 124 | 6.1 | 9.4 |
| Cellular carbohydrate catabolism | 124 | 6.1 | 9.4 |
| Energy derivation by oxidation of organic compounds | 259 | 4.0 | 6.9 |
| Fructose and mannose metabolism | 114 | 4.7 | 6.7 |
| Glucose catabolism | 97 | 6.9 | 12.0 |
| Glucose metabolism | 159 | 4.6 | 8.5 |
| Glycolysis | 83 | 7.2 | 11.5 |
| Glycolysis Gluconeogenesis | 121 | 5.5 | 10.9 |
| Hexose catabolism | 97 | 6.9 | 12.0 |
| Hexose metabolism | 216 | 3.5 | 6.5 |
| Main pathways of carbohydrate metabolism | 185 | 4.8 | 7.4 |
| Monosaccharide catabolism | 97 | 6.9 | 12.0 |
| Monosaccharide metabolism | 218 | 3.6 | 6.6 |
| Oxidoreductase activity, acting on the CH-OH group of donors, NAD or NADP as acceptor | 164 | 3.8 | 3.2 |
| Pentose phosphate pathway | 55 | 6.1 | 8.5 |
| **Lipid** |  |  |  |
| Biosynthesis of steroids | 30 | 6.3 | 12.6 |
| C21-steroid hormone biosynthesis | 33 | 3.6 | 4.7 |
| C21-steroid hormone metabolism | 41 | 4.0 | 3.5 |
| Carboxylic acid biosynthesis | 116 | 3.3 | 5.4 |
| Cholesterol biosynthesis | 47 | 10.7 | 18.7 |
| Cholesterol metabolism | 112 | 8.7 | 11.6 |
| Fatty acid biosynthesis | 107 | 3.3 | 5.7 |
| Hormone biosynthesis | 48 | 4.2 | 5.4 |
| Lipid biosynthesis | 396 | 7.2 | 11.8 |
| Organic acid biosynthesis | 116 | 3.3 | 5.4 |
| Steroid biosynthesis | 120 | 9.8 | 15.8 |
| Steroid metabolism | 234 | 8.4 | 10.7 |
| Sterol biosynthesis | 54 | 11.5 | 19.5 |
| Sterol metabolism | 121 | 9.3 | 12.5 |
| **Ribosome** |  |  |  |
| Cytosolic ribosome (sensu Eukaryota) | 125 | -7.5 | -4.7 |
| Ribosome | 381 | -7.0 | -6.7 |
| Ribosome | 215 | -10.3 | -7.1 |
| Ribosome biogenesis | 199 | -4.2 | -9.4 |
| Ribosome biogenesis and assembly | 213 | -5.0 | -9.2 |
| structural constituent of ribosome | 440 | -6.5 | -6.7 |
| **Others** |  |  |  |
| Branching morphogenesis | 76 | 4.1 | -3.3 |
| Carbon fixation | 53 | 4.4 | 7.1 |
| Cytoplasm organization and biogenesis | 243 | -5.4 | -8.8 |
| JAK STAT Signaling Pathway | 230 | -5.9 | -4.6 |
| Tube morphogenesis | 116 | 5.9 | -3.4 |
| Unlocalized protein complex | 128 | 3.8 | -3.5 |
| Vasculature development | 267 | 3.7 | -3.3 |
