# Supplementary material for: Unbiased Gene Expression Analysis Implicates the huntingtin Polyglutamine Tract in Extra-mitochondrial Energy Metabolism
Source: PLoS Genet. 2007 Aug 17;3(8):e135. doi: 10.1371/journal.pgen.0030135 (PMC1950164; doi:10.1371/journal.pgen.0030135)
Supplement: Supplementary file 6 [file pgen.0030135.st010.doc]

**Supplementary Table 10. *HD* CAG-specific gene sets (Group 1)**

Significantly enriched gene sets in *HD* CAG and 3-NP comparison were identified by gene set analysis (sigPathway). 114 and 196 gene sets were significantly enriched in *HD* CAG and 3-NP model, respectively (q<0.01). Among 266 significant gene sets, 47 gene sets were *HD* CAG-specific (*HD* CAG, q<0.01 and 3-NP, q≥0.05). Size, number of probes in the gene set. NT_k_, normalized gene set score obtained by gene set permutation.

| **Group 1. *HD* CAG-specific Gene Set** | Size | NT_k_ | |
| --- | --- | --- | --- |
|  |  | *HD* CAG | 3-NP |
| **Development / Morphogenesis / Growth** |  |  |  |
| Angiogenesis | 218 | -5.9 | 0.3 |
| Apical part of cell | 95 | 4.0 | 0.1 |
| Apical plasma membrane | 55 | 4.3 | -0.7 |
| Brain development | 169 | 3.9 | 0.9 |
| Cell growth | 156 | -6.9 | -2.1 |
| Cell migration | 433 | 7.1 | -1.1 |
| Cell projection | 404 | 5.2 | -1.3 |
| Cell surface | 225 | 3.3 | 0.1 |
| Growth | 258 | -7.5 | -2.3 |
| Growth factor binding | 103 | -8.2 | -2.0 |
| Mesoderm development | 64 | 3.7 | 2.0 |
| Osteogenesis | 215 | -8.2 | 1.3 |
| Regulation of cell migration | 85 | 4.6 | 0.3 |
| Regulation of cell motility | 89 | 4.7 | 0.8 |
| Regulation of cell size | 166 | -6.8 | -1.9 |
| Regulation of growth | 187 | -4.4 | -1.9 |
| TGF BMP Signaling Pathway | 216 | -3.5 | 0.1 |
| Ureteric bud development | 34 | 4.1 | -1.8 |
| **Neuronal** |  |  |  |
| Axon | 49 | 4.8 | 0.5 |
| Axon guidance | 146 | 6.4 | -1.8 |
| Axonogenesis | 241 | 6.2 | -0.8 |
| Central nervous system development | 204 | 3.6 | 1.8 |
| Dendrite | 41 | 6.8 | 0.7 |
| Forebrain development | 98 | 4.1 | 2.2 |
| Learning and/or memory | 78 | 4.5 | -0.7 |
| Neurite morphogenesis | 292 | 6.4 | -1.0 |
| Neuron migration | 98 | 4.0 | 1.7 |
| Neuron projection | 92 | 5.9 | 0.8 |
| Regulation of neurogenesis | 62 | 4.5 | 0.8 |
| Synapse | 333 | 4.8 | 1.9 |
| Synaptic vesicle | 112 | 5.4 | -0.2 |
| **Others** |  |  |  |
| Th1-Th2-Th3 | 212 | -3.3 | -2.3 |
| Anion transport | 277 | -3.4 | 0.8 |
| Cell adhesion molecules CAMs | 340 | 5.7 | -0.6 |
| Clathrin-coated vesicle | 193 | 3.3 | -0.5 |
| Connexon channel activity | 28 | -3.3 | -1.1 |
| Connexon complex | 28 | -3.3 | -1.1 |
| Extracellular matrix structural constituent | 112 | -8.4 | 1.3 |
| Fibroblast growth factor receptor binding | 48 | -3.7 | -0.1 |
| Gap-junction forming channel activity | 28 | -3.3 | -1.1 |
| Inorganic anion transport | 241 | -3.6 | 1.0 |
| Oxidoreductase activity, acting on the CH-NH2 group of donors, oxygen as acceptor | 25 | -3.3 | 0.1 |
| Phosphate transport | 98 | -10.2 | 1.2 |
| Regulation of behavior | 98 | 4.6 | 0.8 |
| Regulation of blood pressure | 25 | 3.4 | 1.2 |
| Regulation of locomotion | 97 | 4.7 | 0.8 |
| Response to wounding | 415 | -3.5 | -0.2 |
