# Supplementary material for: Unbiased Gene Expression Analysis Implicates the huntingtin Polyglutamine Tract in Extra-mitochondrial Energy Metabolism
Source: PLoS Genet. 2007 Aug 17;3(8):e135. doi: 10.1371/journal.pgen.0030135 (PMC1950164; doi:10.1371/journal.pgen.0030135)
Supplement: Supplementary file 7 [file pgen.0030135.st011.doc]

**Supplementary Table 11. 3-NP-specific gene sets (Group 5)**

Significantly enriched gene sets in *HD* CAG and 3-NP comparison were identified by gene set analysis (sigPathway). 114 and 196 gene sets were significantly enriched in *HD* CAG and 3-NP model, respectively (q<0.01). Among 266 significant gene sets, 117 gene sets were 3-NP-specific (*HD* CAG, q≥0.05 and 3-NP, q<0.01). Size, number of probes in the gene set. NT_k_, normalized gene set score obtained by gene set permutation.

| **Group 5. 3-NP-specific Gene Set** | Size | NT_k_ | |
| --- | --- | --- | --- |
|  |  | *HD* CAG | 3-NP |
| **Cellular metabolism** |  |  |  |
| Alanine and aspartate metabolism | 45 | -1.0 | -5.5 |
| Amine biosynthesis | 104 | 2.3 | -5.2 |
| Amino acid and derivative metabolism | 450 | 0.5 | -4.7 |
| Amino acid biosynthesis | 72 | 2.0 | -5.3 |
| Amino acid metabolism | 351 | 0.6 | -5.6 |
| Amino sugar metabolism | 48 | -0.9 | -3.4 |
| Aminosugars metabolism | 69 | -1.4 | -3.3 |
| Arginine and proline metabolism | 71 | -2.0 | -3.5 |
| Butanoate metabolism | 103 | 2.1 | 3.2 |
| C-acyltransferase activity | 24 | 1.0 | 3.3 |
| Fatty acid metabolism | 262 | 2.1 | 4.7 |
| Glucosamine metabolism | 35 | -1.3 | -3.5 |
| Glutamate metabolism | 55 | -1.0 | -3.5 |
| Glutamine family amino acid metabolism | 36 | -1.0 | -3.1 |
| N-acetylglucosamine metabolism | 35 | -1.3 | -3.5 |
| NADP metabolism | 20 | 2.2 | 5.8 |
| Nicotinamide metabolism | 33 | 2.0 | 3.4 |
| Nitrogen compound biosynthesis | 104 | 2.3 | -5.2 |
| Nucleobase metabolism | 29 | -1.3 | -3.3 |
| One carbon pool by folate | 40 | -0.2 | -3.5 |
| Oxidoreduction coenzyme metabolism | 44 | 2.2 | 3.2 |
| Pyridine nucleotide metabolism | 40 | 1.9 | 3.3 |
| S-adenosylmethionine-dependent methyltransferase activity | 162 | -0.5 | -3.7 |
| Urea cycle and metabolism of amino groups | 37 | -1.8 | -3.7 |
| **Cell cycle** |  |  |  |
| BTG family proteins and cell cycle regulation | 29 | -0.9 | -3.3 |
| Chromosome segregation | 74 | 1.5 | 5.9 |
| Influence of Ras and Rho proteins on G1 to S Transition | 71 | -0.9 | -4.1 |
| Interphase | 138 | -0.6 | -3.2 |
| Interphase of mitotic cell cycle | 138 | -0.6 | -3.2 |
| M phase | 336 | 0.0 | 5.4 |
| M phase of meiotic cell cycle | 90 | 0.6 | 3.8 |
| M phase of mitotic cell cycle | 262 | 0.0 | 4.2 |
| Meiosis | 90 | 0.6 | 3.8 |
| Meiotic cell cycle | 90 | 0.6 | 3.8 |
| Mitosis | 261 | 0.1 | 4.5 |
| Polyamine metabolism | 32 | 0.0 | -3.1 |
| **Structure molecule / Adhesion** |  |  |  |
| Actin cytoskeleton | 458 | 0.8 | -3.4 |
| Adhesion Molecules on Lymphocyte | 22 | 2.0 | -3.4 |
| Cell-matrix adhesion | 130 | 0.9 | -3.1 |
| Extracellular Matrix Adhesion Molecules | 218 | 0.2 | -5.1 |
| Focal adhesion | 61 | 2.4 | -3.5 |
| Microtubule | 362 | 1.5 | 3.2 |
| **Signaling** |  |  |  |
| ATM Signaling Pathway | 55 | -2.4 | -3.8 |
| Breast Cancer Estrogen Receptor Signaling | 225 | -1.0 | -4.7 |
| Ca NFAT Signaling Pathways | 244 | 0.5 | -3.4 |
| GTPase activator activity | 313 | -2.1 | -3.1 |
| HIV-I Nef negative effector of Fas and TNF | 135 | -1.5 | -6.0 |
| Kinase regulator activity | 139 | 2.4 | -3.3 |
| MAP Kinase Signaling Pathway | 260 | -0.1 | -3.4 |
| Neuroactive ligand-receptor interaction | 428 | 1.5 | 4.1 |
| NFkB Signaling Pathway | 206 | -0.6 | -4.1 |
| P53 Signaling Pathway | 51 | 2.4 | -6.0 |
| P53 Signaling Pathway | 253 | -1.4 | -5.1 |
| Regulation of MAP Kinase Pathways Through Dual Specificity Phosphatases | 20 | 0.5 | -3.2 |
| Rhodopsin-like receptor activity | 452 | 1.3 | 3.8 |
| Signal Transduction in Cancer | 202 | -0.3 | -4.1 |
| Signal Transduction PathwayFinder | 166 | -1.8 | -3.9 |
| Signal transduction through IL1R | 73 | -1.7 | -3.7 |
| Signaling of Hepatocyte Growth Factor Receptor | 104 | 1.4 | -4.0 |
| Small GTPase mediated signal transduction | 498 | -0.8 | -3.3 |
| Stress Toxicity PathwayFinder | 193 | -0.6 | -6.7 |
| **RNA metabolism** |  |  |  |
| RNA splicing | 369 | -0.2 | 5.4 |
| mRNA processing | 480 | -0.3 | 4.4 |
| mRNA splice site selection | 60 | 0.4 | 3.1 |
| Nuclear mRNA splicing, via spliceosome | 349 | -0.7 | 5.1 |
| RNA splicing, via transesterification reactions | 349 | -0.7 | 5.1 |
| RNA splicing, via transesterification reactions with bulged adenosine as nucleophile | 349 | -0.7 | 5.1 |
| rRNA metabolism | 70 | -0.8 | -5.5 |
| rRNA processing | 70 | -0.8 | -5.5 |
| tRNA metabolism | 138 | 0.9 | -6.3 |
| RNA ligase activity | 102 | 1.8 | -6.1 |
| RNA modification | 98 | 2.3 | -6.4 |
| **Enzyme activity** |  |  |  |
| Cancer Drug Resistance Metabolism | 213 | 1.7 | -3.4 |
| Enzyme activator activity | 397 | -1.8 | -3.5 |
| Hydrolase activity, acting on carbon-nitrogen (but not peptide) bonds, in cyclic amidines | 43 | -1.1 | -3.9 |
| Isomerase activity | 231 | -0.5 | 3.3 |
| Ligase activity, forming aminoacyl-tRNA and related compounds | 102 | 1.8 | -6.1 |
| Ligase activity, forming carbon-oxygen bonds | 102 | 1.8 | -6.1 |
| Ligase activity, forming phosphoric ester bonds | 113 | 1.6 | -5.9 |
| Monooxygenase activity | 171 | 0.0 | 6.4 |
| Oxidoreductase activity, acting on paired donors, with incorporation or reduction of molecular oxygen | 148 | 2.4 | 9.9 |
| Oxidoreductase activity, acting on paired donors, with incorporation or reduction of molecular oxygen, 2-oxoglutarate as one donor, and incorporation of one atom each of oxygen into both donors | 35 | 0.3 | 6.4 |
| Oxidoreductase activity, acting on paired donors, with incorporation or reduction of molecular oxygen, NAD or NADH as one donor, and incorporation of one atom of oxygen | 26 | 2.2 | 5.4 |
| Oxidoreductase activity, acting on the CH-NH group of donors, NAD or NADP as acceptor | 23 | -0.3 | -3.2 |
| **Translation / tRNA** |  |  |  |
| Aminoacyl-tRNA synthetases | 40 | 1.4 | -6.7 |
| Translational initiation | 109 | -1.5 | -5.1 |
| Translation factor activity, nucleic acid binding | 250 | -1.8 | -4.9 |
| tRNA ligase activity | 102 | 1.8 | -6.1 |
| tRNA modification | 86 | 1.9 | -6.6 |
| translation regulator activity | 259 | -1.9 | -4.8 |
| tRNA aminoacylation for protein translation | 80 | 2.0 | -6.2 |
| tRNA aminoacylation | 80 | 2.0 | -6.2 |
| Translation initiation factor activity | 165 | -2.3 | -5.4 |
| Translation | 303 | -0.5 | -6.2 |
| **Others** |  |  |  |
| Agrin in Postsynaptic Differentiation | 96 | -0.5 | -3.3 |
| Amino acid activation | 80 | 2.0 | -6.2 |
| Aangiogenesis | 192 | 2.3 | -3.9 |
| Cancer PathwayFinder | 235 | 1.0 | -6.7 |
| Cardiovascular Disease | 309 | 0.3 | -4.7 |
| Chromosome, pericentric region | 89 | -0.7 | 3.7 |
| Inner membrane | 291 | -0.6 | -3.1 |
| Integrin binding | 30 | -1.5 | -3.6 |
| Iron ion binding | 419 | 2.0 | 5.7 |
| Kinetochore | 47 | 0.7 | 3.3 |
| L-ascorbic acid binding | 25 | -1.0 | 6.1 |
| Late endosome | 51 | 1.1 | -4.7 |
| Monocyte and its Surface Molecules | 26 | 1.9 | -3.3 |
| Nucleolus | 185 | -0.8 | -5.6 |
| Protein targeting | 482 | -0.7 | -3.2 |
| Protein transporter activity | 326 | -1.9 | -4.0 |
| Regulation of gene expression, epigenetic | 107 | 0.8 | 3.3 |
| Sex differentiation | 103 | 0.1 | 3.2 |
| Small ribosomal subunit | 89 | -2.1 | -3.2 |
| Transcriptional activator activity | 163 | -2.4 | -4.2 |
| Tumor Metastasis | 220 | 1.3 | -7.5 |
| Tumor Suppressor Arf Inhibits Ribosomal Biogenesis | 31 | -0.8 | -3.3 |
| uCalpain and friends in Cell spread | 39 | -0.2 | -3.3 |
