# Supplementary material for: Unbiased Gene Expression Analysis Implicates the huntingtin Polyglutamine Tract in Extra-mitochondrial Energy Metabolism
Source: PLoS Genet. 2007 Aug 17;3(8):e135. doi: 10.1371/journal.pgen.0030135 (PMC1950164; doi:10.1371/journal.pgen.0030135)
Supplement: Supplementary file 8 [file pgen.0030135.st012.doc]

Supplementary Table 12. Significant pathways identified by both GO analysis and gene set analysis

Significantly altered GO terms (DAVID 2006) were compared to significantly enriched gene sets (sigPathway) to identify significant pathways called by two different pathway analysis algorithms. %, % of probes with defined gene ontology term in the significant probes. p value, Fisher’s exact test p value. NT_k_, normalized gene set score obtained by gene set permutation.

| Pathway (GO term) | GO | | sigPathway | |
| --- | --- | --- | --- | --- |
|  | % | p value | NT_k_ | q value |
| ***HD* CAG : Increase** |  |  |  |  |
| anion transport | 3.19 | 0.0006 | -3.4 | 0.0069 |
| cell growth | 3.19 | 0.0000 | -6.9 | 0.0000 |
| growth | 3.51 | 0.0001 | -7.5 | 0.0000 |
| inorganic anion transport | 3.19 | 0.0002 | -3.6 | 0.0043 |
| phosphate transport | 3.19 | 0.0000 | -10.2 | 0.0000 |
| regulation of cell growth | 2.56 | 0.0001 | -6.8 | 0.0000 |
| regulation of cell size | 3.19 | 0.0000 | -6.8 | 0.0000 |
| regulation of growth | 2.56 | 0.0009 | -4.4 | 0.0016 |
| regulation of signal transduction | 2.88 | 0.0038 | -3.4 | 0.0069 |
| response to wounding | 4.15 | 0.0145 | -3.5 | 0.0063 |
| ***HD* CAG : Decrease** |  |  |  |  |
| alcohol catabolism | 1.51 | 0.0450 | 6.9 | 0.0000 |
| alcohol metabolism | 4.91 | 0.0000 | 8.5 | 0.0000 |
| axonogenesis | 2.26 | 0.0186 | 6.2 | 0.0000 |
| brain development | 2.26 | 0.0109 | 3.9 | 0.0009 |
| cell migration | 6.04 | 0.0000 | 7.1 | 0.0000 |
| cell-cell adhesion | 2.26 | 0.0500 | 5.4 | 0.0000 |
| central nervous system development | 2.64 | 0.0060 | 3.6 | 0.0036 |
| cholesterol biosynthesis | 2.26 | 0.0000 | 10.7 | 0.0000 |
| cholesterol metabolism | 2.26 | 0.0005 | 8.7 | 0.0000 |
| forebrain development | 1.51 | 0.0233 | 4.1 | 0.0014 |
| glucose catabolism | 1.51 | 0.0417 | 6.9 | 0.0000 |
| glucose metabolism | 2.26 | 0.0065 | 4.6 | 0.0000 |
| hexose catabolism | 1.51 | 0.0433 | 6.9 | 0.0000 |
| hexose metabolism | 2.26 | 0.0192 | 3.5 | 0.0061 |
| lipid biosynthesis | 4.15 | 0.0001 | 7.2 | 0.0000 |
| monosaccharide catabolism | 1.51 | 0.0433 | 6.9 | 0.0000 |
| monosaccharide metabolism | 2.26 | 0.0203 | 3.6 | 0.0046 |
| neurite morphogenesis | 2.64 | 0.0099 | 6.4 | 0.0000 |
| regulation of angiogenesis | 1.13 | 0.0386 | 3.5 | 0.0058 |
| regulation of cell migration | 1.51 | 0.0233 | 4.6 | 0.0009 |
| regulation of cell motility | 1.51 | 0.0258 | 4.7 | 0.0009 |
| regulation of locomotion | 1.51 | 0.0297 | 4.7 | 0.0009 |
| steroid biosynthesis | 3.02 | 0.0000 | 9.8 | 0.0000 |
| steroid metabolism | 3.02 | 0.0011 | 8.4 | 0.0000 |
| sterol biosynthesis | 2.64 | 0.0000 | 11.5 | 0.0000 |
| sterol metabolism | 2.64 | 0.0001 | 9.3 | 0.0000 |
| tube morphogenesis | 2.26 | 0.0020 | 5.9 | 0.0000 |
| vasculature development | 4.53 | 0.0000 | 3.7 | 0.0032 |
| **3-NP : Increase** |  |  |  |  |
| amine biosynthesis | 0.99 | 0.0104 | -5.2 | 0.0000 |
| amino acid and derivative metabolism | 2.69 | 0.0010 | -4.7 | 0.0005 |
| amino acid metabolism | 1.98 | 0.0125 | -5.6 | 0.0000 |
| angiogenesis | 1.27 | 0.0171 | -3.9 | 0.0011 |
| Apoptosis | 3.96 | 0.0015 | -2.7 | 0.0208 |
| blood vessel development | 1.41 | 0.0395 | -3.5 | 0.0040 |
| blood vessel morphogenesis | 1.27 | 0.0446 | -3.2 | 0.0071 |
| cytoplasm organization and biogenesis | 1.98 | 0.0001 | -8.8 | 0.0000 |
| nitrogen compound biosynthesis | 0.99 | 0.0104 | -5.2 | 0.0000 |
| regulation of cell growth | 0.99 | 0.0334 | -2.5 | 0.0321 |
| ribosome biogenesis | 1.84 | 0.0000 | -9.4 | 0.0000 |
| ribosome biogenesis and assembly | 1.84 | 0.0001 | -9.2 | 0.0000 |
| small GTPase mediated signal transduction | 2.12 | 0.0209 | -3.3 | 0.0053 |
| translation | 1.56 | 0.0206 | -6.2 | 0.0000 |
| vasculature development | 1.41 | 0.0437 | -3.3 | 0.0053 |
| **3-NP : Decrease** |  |  |  |  |
| alcohol catabolism | 2.55 | 0.0000 | 11.9 | 0.0000 |
| alcohol metabolism | 7.32 | 0.0000 | 12.5 | 0.0000 |
| carbohydrate catabolism | 2.55 | 0.0002 | 9.4 | 0.0000 |
| carboxylic acid biosynthesis | 1.59 | 0.0104 | 5.4 | 0.0000 |
| cellular carbohydrate catabolism | 2.55 | 0.0002 | 9.4 | 0.0000 |
| cellular carbohydrate metabolism | 3.82 | 0.0023 | 3.6 | 0.0028 |
| cholesterol biosynthesis | 4.14 | 0.0000 | 18.7 | 0.0000 |
| cholesterol metabolism | 4.14 | 0.0000 | 11.6 | 0.0000 |
| chromatin assembly | 2.55 | 0.0002 | 5.3 | 0.0000 |
| chromatin assembly or disassembly | 2.55 | 0.0009 | 3.5 | 0.0030 |
| chromosome organization and biogenesis | 3.82 | 0.0061 | 3.2 | 0.0077 |
| coenzyme metabolism | 2.55 | 0.0183 | 3.3 | 0.0053 |
| cofactor metabolism | 2.87 | 0.0112 | 3.3 | 0.0065 |
| DNA packaging | 4.14 | 0.0001 | 3.2 | 0.0077 |
| establishment and/or maintenance of chromatin architecture | 3.82 | 0.0002 | 3.2 | 0.0074 |
| fatty acid biosynthesis | 1.59 | 0.0087 | 5.7 | 0.0000 |
| glucose catabolism | 2.55 | 0.0000 | 12.0 | 0.0000 |
| glucose metabolism | 2.87 | 0.0001 | 8.5 | 0.0000 |
| glycolysis | 2.23 | 0.0002 | 11.5 | 0.0000 |
| hexose catabolism | 2.55 | 0.0000 | 12.0 | 0.0000 |
| hexose metabolism | 2.87 | 0.0005 | 6.5 | 0.0000 |
| isoprenoid metabolism | 1.59 | 0.0003 | 6.8 | 0.0000 |
| lipid biosynthesis | 7.32 | 0.0000 | 11.8 | 0.0000 |
| M phase | 2.87 | 0.0050 | 5.4 | 0.0000 |
| M phase of mitotic cell cycle | 2.23 | 0.0104 | 4.2 | 0.0006 |
| microtubule-based process | 2.23 | 0.0450 | 3.5 | 0.0033 |
| mitosis | 2.23 | 0.0100 | 4.5 | 0.0000 |
| monosaccharide catabolism | 2.55 | 0.0000 | 12.0 | 0.0000 |
| monosaccharide metabolism | 2.87 | 0.0006 | 6.6 | 0.0000 |
| mRNA processing | 2.55 | 0.0163 | 4.4 | 0.0000 |
| nuclear mRNA splicing, via spliceosome | 1.91 | 0.0312 | 5.1 | 0.0000 |
| nucleosome assembly | 2.55 | 0.0001 | 6.7 | 0.0000 |
| organic acid biosynthesis | 1.59 | 0.0104 | 5.4 | 0.0000 |
| protein complex assembly | 3.18 | 0.0004 | 5.3 | 0.0000 |
| RNA splicing | 2.23 | 0.0108 | 5.4 | 0.0000 |
| RNA splicing, via transesterification reactions | 1.91 | 0.0312 | 5.1 | 0.0000 |
| RNA splicing, via transesterification reactions with bulged adenosine as nucleophile | 1.91 | 0.0312 | 5.1 | 0.0000 |
| steroid biosynthesis | 5.41 | 0.0000 | 15.8 | 0.0000 |
| steroid metabolism | 5.41 | 0.0000 | 10.7 | 0.0000 |
| sterol biosynthesis | 4.46 | 0.0000 | 19.5 | 0.0000 |
| sterol metabolism | 4.46 | 0.0000 | 12.5 | 0.0000 |
