# Supplementary material for: Unbiased Gene Expression Analysis Implicates the huntingtin Polyglutamine Tract in Extra-mitochondrial Energy Metabolism
Source: PLoS Genet. 2007 Aug 17;3(8):e135. doi: 10.1371/journal.pgen.0030135 (PMC1950164; doi:10.1371/journal.pgen.0030135)
Supplement: Supplementary file 9 [file pgen.0030135.st013.doc]

# Supplementary Table 13. Primers sets for gene specific PCR assays

| **Mitochondrial genome gene expression analysis** | | |
| --- | --- | --- |
| ND1 | CAACCCTAGCAGAAACAAACC | CGGCTGCGTATTCTACGTTA |
| ND2 | GGAAGGCCTCCTAGGGATAG | AACCATCAACTCAATCTCACTTCT |
| ND3 | GGGCTAGTCCTACAGCTGCTT | TCGGTAACTTCCCTAAACTCCA |
| ND4 | ACATGGCCTCACATCATCAC | AGTCCTCGGGCCATGATTA |
| ND5 | TCACCGCATTACACCCATAA | GGTGGATTTTGGGATGGTTT |
| COX1 | TAGCGTCGTGGTATTCCTGA | GATTCCCATTATTTTCAGGCTTC |
| COX2 | ACCGAGTCGTTCTGCCAATA | AAGTCCTAGGGAGGGGACTG |
| COX3 | TGGAATCCAGTAGCCATGAA | TTACTTCACCATCCTCCAAGC- |
| CYTB | ACCCCACCCCATATTAAACC | GGCTAGGACACCTCCTAGTTTATTG |
| β-Actin | GACGGCCAGGTCATCACTAT | ATGCCACAGGATTCCATACC |
|  |  |  |
| **Nrf-1 target gene expression analysis** | | |
| Ab*HD*5 | AATGCTTCAGCGGATAGGTG | AGTGACTGGATGCTGGTTCC |
| Adssl1 | AACACTGCCTGGATGGAAAG | TGCAACACCCATGTGATTCT |
| Akap8 | CTCTGCACCAGTTTGTTCCA | CAGCTATGCCAACAGCAGAC |
| Arpc1a | AGCGCAACATGTCTGCTATG | TCTGGTGCAGTGTTTCCAAG |
| Atp6v1g1 | TTGACCCAGATGTTGAGCAG | CCCAAATGAACACCCTGAGT |
| Cxcr4 | TGGTGGTGTTTCAATTCCAG | CCCTTGGAGTGTGACAGCTT |
| Dctn6 | CCGGATAACATCATCCCAGA | TCTCCCATTTTCATGGCTTG |
| Hspca | GTGTTCTCGGGACTTTCCAA | ATTCGGCCGGTTTTAGTCTT |
| Nfib | CTTGCTTCCCAGTCCTCATT | GGATAAGGGCCAAGGAGGT |
| Rpl10a | AGCAGATCCCACGTCTCCTG | TGTCGATTTCACCTCATCCA |
| Sfrs2 | GTCCTCCTCCGTCTCCAGAT | AGACTTGGACTCTCGCTTCG |
| Smad5 | CCCACCACCGTCTGTAAGAT | CCTCGAATCCATGGTTGACT |
| Stag1 | TCTTGTTCAAGCAGCCACTG | CCACTCCATGGAATTGGAAG |
| Uble1a | GCACTGTTACCCAGCTCCTT | AAGGGTGCCCTCATACTCCT |
|  |  |  |
| **Microarray experiment verification** | | |
| β-Actin | TGTTCACAACTGGGACGACA | CTCTCAGCTGTGGTGGTGAA |
| Ak5 | AAGGCACCCAGTGTGAGAAG | CTTGCTCTGGTGTTCCCTCT |
| Aldoa | GAACCAATGGCGAGACAACT | GGGGCACTTCTTGATAGCAT |
| Aqp5 | CTGGGACCTGTGAGTGGTG | CGAGGGGAAAAGCAAGTAGAA |
| Ckb | GCTCATTGACGACCACTTCC | AGTTTCACTCCGTCCACCAC |
| Ctsz | AATGCTGGCTCTTGTGAAGG | ACTCTCGATGGCAAGGTTGT |
| Gtl2 | TTGCTGTTGTGCTCAGGTTC | CCTGAGCCCATTTCACAGAT |
| Ldh1 | AGCTGCTGATCGTCTCCAAT | TCACAACATCCGAGATTCCA |
| Ldh2 | CCTCCAGACTCCGAAAATTG | TTCATAGGCACTGTCCACCA |
| Olfm1 | CGTCCGTGAGTACAAGTCCA | GTTCTGGAAGGGGATGTCAA |
| Pcx | GAGCCCTTTCGCTCTAAGGT | TGCTGCCACCTTGATGTCTA |
| Pdfgra | GGTCCTATGTCATTCTGCTC | TCCGAAGTCTGTGAGCTGTG |
| Pfkl | CGAGGAACTCTGCATTGTCA | CTCAGACACCCACAACATGG |
| Pkp1 | TGCCTACACCGAGAAGTCCT | GGCTGTGAGGTCATCAGGTT |
| Tagln | GGAGCGACTAGTGGAGTGGA | ATTTGAAGGCCAATGACGTG |
| Tgfbi | ACGCCCAGATGAAGACTTTG | CAGTTTGTCCCCTTGGAGAG |
| Tpi1 | CATCGACTTTGCCAGACAGA | CAGGTTGCTCCAGTCACAGA |
